# The Tetraspanins CD9 and CD81 Regulate CD9P1-Induced Effects on Cell Migration

**DOI:** 10.1371/journal.pone.0011219

**Published:** 2010-06-21

**Authors:** Célia Chambrion, François Le Naour

**Affiliations:** 1 Inserm U1004, Villejuif, France; 2 Univ. Paris-Sud 11, Institut André Lwoff, Villejuif, France; 3 Inserm U785, Villejuif, France; Emory Unviersity, United States of America

## Abstract

CD9P-1 is a cell surface protein with immunoglobulin domains and an unknown function that specifically associates with tetraspanins CD9 and CD81. Overexpression of CD9P-1 in HEK-293 cells induces dramatic changes in cell spreading and migration on various matrices. Experiments using time-lapse videomicroscopy revealed that CD9P-1 expression has led to higher cell motility on collagen I but lower motility on fibronectin through a β1-integrins dependent mechanism. On collagen I, the increase in cell motility induced by CD9P-1 expression was found to involve integrin α2β1 and CD9P-1 was observed to associate with this collagen receptor. The generation of CD9P-1 mutants demonstrated that the transmembrane and the cytoplasmic domains are necessary for inducing effects on cell motility. On the other hand, expression of tetraspanins CD9 or CD81 was shown to reverse the effects of CD9P-1 on cell motility on collagen I or fibronectin with a concomitant association with CD9P-1. Thus, the ratio of expression levels between CD9P-1 and its tetraspanin partners can regulate cell motility.

## Introduction

Tetraspanins are integral membrane proteins characterized by significant sequence identity and specific structural features [1]–[3]. They are highly expressed on many cell types and have been involved in a large variety of physiological and pathological processes such as immune response, reproduction and development, infectious and genetic diseases as well as metastasis [1]–[9]. For example, it has been demonstrated using knock-out mice that the tetraspanin CD9 plays an essential role in reproduction because CD9 deficient mice exhibited severely reduced female fertility because of impaired gamete fusion [10]. Another tetraspanin has been shown to be involved in the immune response as CD81 knock-out mice exhibit an impaired immune response [1], [11]. On the other hand, this tetraspanin has been identified as a receptor involved in hepatitis C virus infection [12]. Similarly, CD81 expression is necessary for *Plasmodium falciparum* entry into hepatocytes [4]. The tetraspanin CD151 has been described to be essential for the correct assembly of human basement membranes in kidney and skin [6], [13], [14].

At the molecular level, tetraspanins associate with each other as well as with numerous other membrane proteins in particular microdomains on the plasma membrane [9], [15]. Several tetraspanins such as CD9, CD63, CD81 and CD151 have been shown to associate with one or a few specific molecular partners, forming small primary complexes [15], [16]. CD9 associates directly with the membrane precursor proHB-EGF (heparin-binding epidermal growth factor) [17], [18] and EpCAM (epithelial cell adhesion molecule) [19], while CD81 associates with the signaling molecule CD19 [20] and integrin α4β1 [16]. In addition, both tetraspanins CD9 and CD81 have been shown to specifically associate with two molecules with immunoglobulin (Ig) domains, CD9P-1 and EWI-2 [2], [21]–[25]. Likewise, the tetraspanin CD63 associates specifically with H,K-ATPase [26] whereas CD151 associates directly with the integrins α3β1, α6β1, α7β1 and α6β4 [2], [16], [27].

Tetraspanins may regulate the expression and trafficking of their molecular partners. CD81, which was initially described as a component of the CD21/CD19/Leu13 complex involved in B-lymphoid cell activation [1], has been demonstrated to be necessary for the expression of its partner CD19 at cell surface of B-lymphocytes [1], [28]. In the absence of CD81, a major reduction of CD19 expression level was observed at cell surface that was correlated with CD19 retention in the endoplasmic reticulum [28], [29]. The tetraspanin CD63 by associating with H,K-ATPase beta subunit allows for its internalization into stomach parietal cells [26]. In contrast, downregulation of CD151 expression significantly slows the internalization rate of integrin α3β1 in cells plated on its main ligand, laminin-5 [30].

Tetraspanins have also been reported to regulate the activity and function of their associated molecules. For example, CD9 expression has been described to increase the binding of diphtheria toxin (DT) on its receptor proHB-EGF, which is associated with CD9 [17]. The increase of DT-sensitivity of the cells was correlated with a higher number of functional binding sites on the receptor, most likely induced by conformational changes [17]. In addition, CD9 expression regulates the juxtacrine activity of membrane-bound HB-EGF factor [31]. It has been demonstrated that CD81 facilitates adhesion of leukocytes on VCAM-1 by increasing the avidity of integrin α4β1 toward its substrate VCAM-1 [32]. CD151 stabilizes the active conformation of integrin α3β1 [33]. Thus, by modulating the ligand-binding activity of integrin α3β1 [33] or α6β1 [34], CD151 markedly influences cell spreading and cellular morphogenesis [35], [36]. Hence, it has been suggested that primary complexes constitute functional units.

In this report, we focus on the functional relevance of primary complexes formed by tetraspanins CD9 or CD81 with CD9P-1. Given that some tetraspanins play a major role in metastasis [8], in particular CD9 which has been reported as a metastasis suppressor, we have addressed the function of CD9P-1 on cell migration that is an important process in metastasis. We show that CD9P-1 overexpression induces dramatic changes in cell migration. We further demonstrate that CD9 and CD81 regulate CD9P1-induced effects on cell motility.

## Materials and Methods

### Cell culture and matrices

The cell line HEK-293 (human embryonic kidney) was obtained from the ATCC. Cell lines stably expressing wild type or mutant CD9P-1 have been described elsewhere [37], [38]. HEK-293 was transfected with pCDNA3 vector (Invitrogen) to establish the cell line HEK-293 mock. All cell lines were cultured in DMEM supplemented with 10% FCS, 2 mM glutamine and antibiotics (all from Invitrogen, Cergy-Pontoise, France). Cells were maintained in a 37°C humidified incubator in the presence of 5% CO_2_. Cells were culture on plastic or on matrices after coating with 15 µg/ml poly-L-ornithine (Sigma), 50 µg/ml collagen I (BD Bioscience), 100 µg/ml matrigel (BD Bioscience), 50 µg/ml laminin-5 or 50 µg/ml fibronectin (Roche).

### Plasmids, transfection and RNA silencing

Encoding cDNA sequence for CD9P-1, CD9, CD81, CD82, CD151 or chimeric molecules CD9×82, CD9P1-HLA-TM-Cyt, CD9P1-HLA-TM, CD9P1-HLA-Cyt were subcloned in the pCDNA3 vector (Invitrogen) as reported elsewhere [38]. For transfection, 5×10^6^ cells were electroporated in 0.4 ml RPMI at room temperature with 10 µg of cDNA using a gene pulser apparatus (Biorad, Ivry, France). The conditions were 300 V and 500 µF for all cell lines.

For RNA silencing, 1–3×10^5^ cells in DMEM medium were transfected with synthetic siRNA oligonucleotides using Interferin (Ozyme) according to the manufacturer's protocol. Cell culture plates (6, 12 or 24 well) were coated with rat tail collagen I (BD Biosciences). Plates were rinsed in PBS, and 1 µl of the siRNA (10 µM) oligonucleotide mix was added to 96 µl DMEM without serum and 3–5 µl Interferin and then incubated at room temperature in each well for 20 min to allow formation of transfection complexes. Cells detached by trypsin-EDTA were layered at the appropriate concentration in 400 µl DMEM medium with serum (the final concentration of siRNA was 20 nM). The following siRNA were synthesized by Eurogentec (Angers, France), si-CD9P-1: AUGUUCAACCCCCAGCACAdTdT, si-control: ACCUCCUCCAGCUCGCUUAdTdT.

### Antibodies

The monoclonal antibodies used in this study were TS9 (CD9), TS9b (CD9), TS81 (CD81), TS82 (CD82), TS151 (CD151) [19], [24], [39], 1F11 (CD9P-1) [24], 12A12 (CD55) [40]. Antibodies directed against integrins were: β1-integrin, 4B4 (Beckman Coulter), β1-vjf; α1-integrin, FB12 (Millipore); α2-integrin, Gi9 (Sigma), H-293 (Santa Cruz Biotechnology, Santa Cruz, CA).

### Immunofluorescence

For flow cytometry analysis of cell surface molecules, cells were detached using a non-enzymatic solution (Invitrogen), washed and stained with saturating concentrations of primary mAb (10 µg/ml). After washing three times with media, cells were incubated with 10 µg/ml FITC-labeled goat anti-mouse antibody (Beckman Coulter). After washing, cells were fixed with 1% formaldehyde in PBS. All incubations were performed for 30 min at 4°C. The analysis of cell surface staining was performed using a FACScalibur flow cytometer (Becton-Dickinson, San Jose, CA).

For *in situ* labeling, cells were cultured in labtek chambers. Cells were fixed in acetone at −20°C for 20 min followed by three washes with PBS 0.2% BSA, 10% SVF. Immunostaining was performed in the chambers with primary antibodies at 10 µg/ml for 45 min. After three washes, the appropriate fluorochrome coupled secondary antibodies (anti-IgG1 coupled to Alexa 568 and anti-IgG2 coupled to Alexa 488 (Invitrogen)) were incubated at the same concentration. For staining of chromatin, an additional incubation was performed with 300 nM DAPI (Invitrogen) for 5 min. Fluorescent labeling was observed with an optical microscope equipped for epifluorescence (Leica, Heerbrugg, Swiss). Images were acquired using MetaVue (Molecular Devices, Toronto, Canada).

### Immunoprecipitation and Western blot

Cells were lysed directly in the flask in the lysis buffer (10 mM Tris pH 7.4, 150 mM NaCl, 0.02% NaN_3_, 1 mM CaCl_2_ and 1 mM MgCl_2_) containing 1% of appropriate detergent (Brij97 or Brij58) (Sigma, St Louis, MO) and proteases inhibitors. After 30 min at 4°C, insoluble material was removed by centrifugation at 12,000 g and the cell lysate was precleared for 2h by addition of 1/1000 volume heat inactivated goat serum and 20 µl protein G sepharose beads (Amersham Bioscience). Proteins were then immunoprecipitated by adding 2 µg of specific antibody and 10µl protein G sepharose beads to 200–400 µl lysate. After a 2h incubation at 4°C under constant agitation, the beads were washed five times in lysis buffer containing 1% of appropriate detergent. The immunoprecipitates were separated by 5–15% SDS-polyacrylamide gel electrophoresis under reducing or non-reducing conditions and transferred to a PVDF or nitrocellulose membrane (Amersham Bioscience). Western blotting on immunoprecipitates was performed using specific mAbs. With PVDF membranes, the revelation was realized by enhanced chemiluminescence (NEN, Boston, MA) after incubation with a streptavidin-biotinylated horseradish peroxidase complex (Amersham Bioscience) when the primary antibody was coupled with biotin; otherwise a secondary goat anti-mouse antibody coupled to horseradish peroxidase (Amersham Bioscience) was used. Proteins on nitrocellulose membranes were revealed with Alexa Fluor 680-labelled streptavidin (Invitrogen) or an Alexa Fluor 680-labelled secondary goat anti-mouse antibody (Invitrogen) using the Odyssey Infrared Imaging System (LI-COR Biosciences).

### Quantitation of stained gels

For quantitative analysis, after SDS-PAGE separation, gels were stained with colloidal Coomassie blue (BioRad) or the Krypton Infrared Protein Stain (Pierce), according to the manufacturer's protocol. Gels were scanned at 700 nm using the Odyssey Infrared Imaging System (LI-COR Biosciences), which allows for quantitation.

### Cell migration assay using time-lapse videomicroscopy or Boyden chambers

Analysis of 2D cell motility was performed using phase contrast on an inverted microscope (Axiovert 200; Zeiss,Oberkochen, Germany) equipped with an environmental chamber with 5% CO2 at 37°C. The microscope was driven by the Metamorph software (Roper Scientific), and images were recorded with a Coolsnap HQ camera (Roper Scientific). Stacks of phase contrast images were collected every 15 min for 24 h at 200× magnification. Cell migration was quantified using the manual tracking plugin of ImageJ [41]. Raw data were transferred to Microsoft Excel for calculations. For each position at least 10 cells were analyzed. Experiments were repeated 2 to 10 times to verify the robustness of the results and the stability of the measurements. For plasmid transfection or silencing experiments, motility measures were performed respectively 24 h and 48 h after treatments.

Cell migration was also investigated using the Boyden chamber assay. In brief, transwell inserts (Corning) with 5 µm pores were coated with collagen I (50 µg/ml) or fibronectin (50 µg/ml). Cells were detached using trypsin and 2×10^4^ cells were plated in the upper chamber and allowed to invade for 18 hours. Noninvasive cells were removed from the upper chamber with a cotton swab, and migrating cells adhering to the underside of the filter were fixed, stained with May Grumwald and Giemsa and enumerated using an optical microscope. A minimum of 5 random fields per filter were counted. All of the experiments were performed independently in duplicate or triplicate.

Statistical analysis of variance (ANOVA) was performed using Microsoft Excel. Results were considered significant when p<0.05.

## Results

### CD9P-1 overexpression induces dramatic changes in cell aggregation and spreading

**Figure 1:**
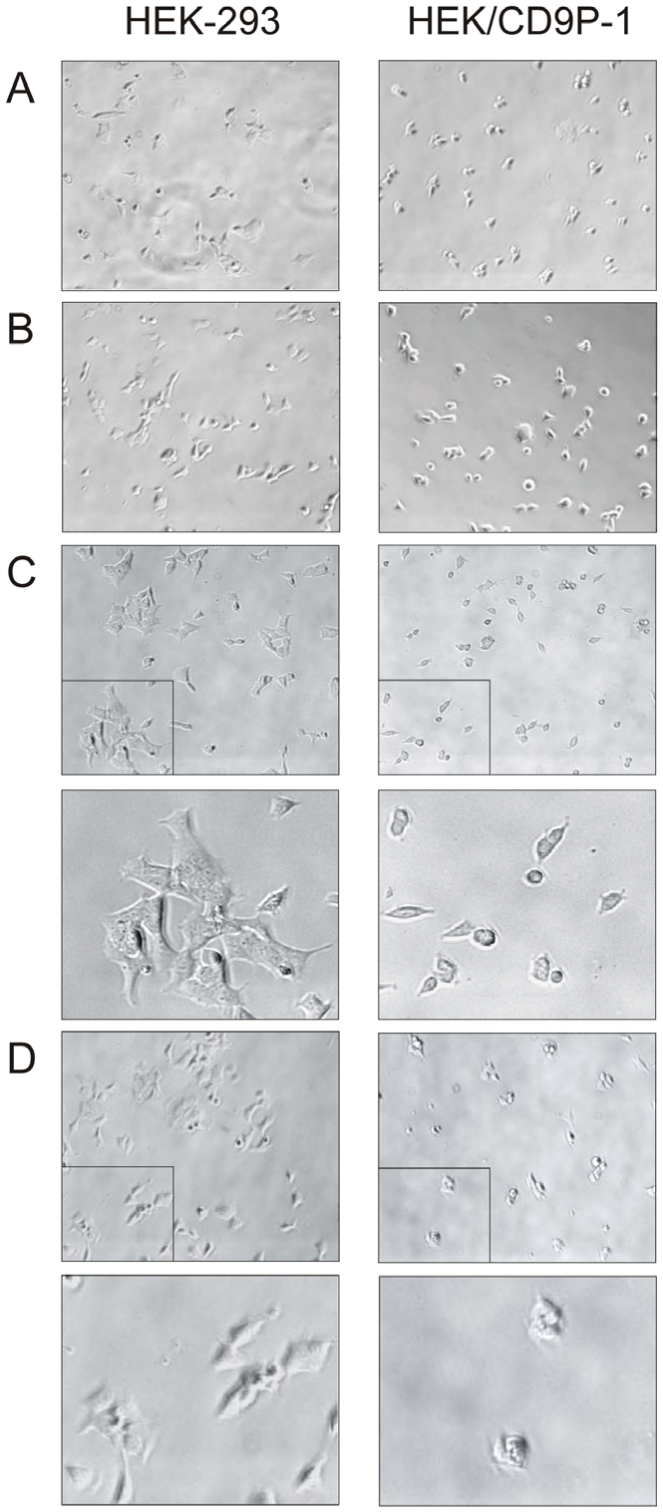
CD9P-1 overexpression affects HEK-293 cell spreading. HEK-293 or HEK/CD9P-1 cells were enzymatically dissociated and plated on various matrices for 24h. (A) Laminin-5. (B) Matrigel. (C) Collagen I. (D) Fibronectin. Cells were photographed with phase contrast (200×). Close up sections are shown with higher magnification (C and D).

To study CD9P-1 function, HEK-293 cells stably expressing CD9P-1 (HEK/CD9P-1) were established and used as a model. The expression levels of tetraspanins (CD9, CD81, CD82 and CD151) and β1-integrins (α1, α2 and β1) were checked at cell surface using flow cytometry. HEK-293 and HEK/CD9P-1 exhibited very similar expression levels (Table 1). The whole stably transfected cell population as well as two selected clones were used for functional assays. Cells were plated on various matrices such as collagen I, laminin-5, fibronectin or matrigel. HEK-293 and HEK/CD9P-1 exhibited different morphology in cell cultured. Indeed, HEK-293 cells formed aggregates after 24h enzymatic dissociation. In contrast, reaggregation and spreading of HEK/CD9P-1 cells were impaired on collagen I and fibronectin (Fig. 1). These observations demonstrate that CD9P-1 expression can dramatically change cell aggregation and spreading.

**Table 1:** Expression of tetraspanins and integrins on HEK-293 and HEK/CD9P-1 cells.

Cell line	CD9	CD81	CD82	CD151	Int. α1	Int. α2	Int. β1
HEK-293	+++	+++	−	+++	+	+	++
HEK/CD9P-1	+++	+++	−	++	+	+	++
HEK/CD9P1-HLA-TM-Cyt	+++	+++	−	+++	+	+	++
HEK/CD9P1-HLA-TM	+++	+++	−	+++	+	+	++
HEK/CD9P1-HLA-Cyt	+++	+++	−	+++	+	+	++
HEK/CD9P-1 Tr CD9	++++						
HEK/CD9P-1 Tr CD81		++++					
HEK/CD9P-1 Tr CD82			++++				
HEK/CD9P-1 Tr CD151				++++			
HEK/CD9P-1 Tr CD9×82			+++				

Fluorescence intensity: −<10; +: 10–50; ++: 50–100; +++: 100–300; ++++>300. Tr: transfection.

### CD9P-1 overexpression induces dramatic changes in cell migration

**Figure 2:**
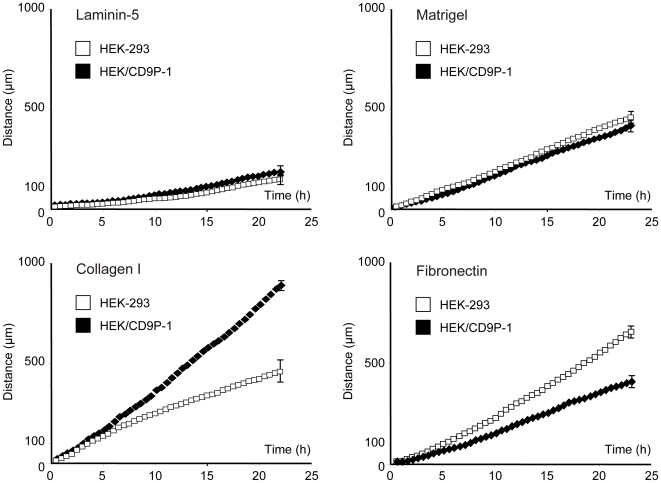
CD9P-1 overexpression affects HEK-293 cell migration. HEK-293 or HEK/CD9P-1 cells were plated on various matrices. The same field of cells was monitored over a period of 24h while being maintained in a microscope stage incubator. Time-lapse videomicroscopy was performed at a rate of 1 frame per 15 min. Independent experiments were performed: laminin-5 (n = 3), matrigel (n = 3), collagen I (n = 20), fibronectin (n = 11); standard errors are shown.

**Figure 3:**
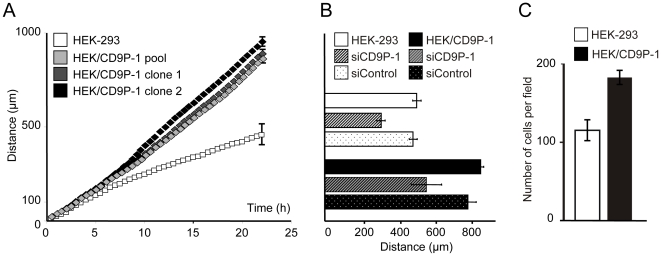
HEK-293 and HEK/CD9P-1 cell migration on collagen I. (A) HEK-293 or HEK/CD9P-1 cells were plated on collagen I. The same field of cells was monitored over a period of 24h while being maintained in a microscope stage incubator. Time-lapse videomicroscopy was performed at a rate of 1 frame per 15 min. Three independent experiments were performed; standard errors are shown. (B) Time-lapse videomicroscopy was performed following CD9P-1 silencing by siRNA. Distances are given over a period of 20h. The results correspond to three independent experiments; standard errors are shown. (C) Cell migration through collagen I was measured using Boyden chambers. Migrating cells adhering to the underside of the filter were enumerated in a minimum of 5 random fields. The results correspond to five independent experiments performed in duplicate or triplicate; standard errors are shown.

The motility of HEK-293 and HEK/CD9P-1 cells was investigated and compared using time-lapse videomicroscopy. Cells were again plated on various matrices such as collagen I, laminin-5, fibronectin or matrigel. Cell migration was monitored for 24h at a rate of 1 frame per 15 min. Hence, tracking individual cells allowed quantifying cell motility. HEK-293 and HEK/CD9P-1 cells exhibited very low motility on laminin-5. On matrigel, both cell lines showed similar motility with a mean rate of 16 µm/h. On fibronectin, HEK-293 exhibited a high motility with a mean rate of 30 µm/h. However, expression of CD9P-1 significantly decreased cell motility on fibronectin to 14 µm/h. Finally, HEK/CD9P-1 cells were observed to be highly motile on collagen I (37 µm/h) compared with HEK-293 cells (16 µm/h) (Fig. 2). This functional effect of CD9P-1 expression on cell migration was observed with the whole stably transfected cell population (pool) as well as two selected clones (Fig. 3A). To confirm that CD9P-1 plays a role in cell motility, silencing experiments were performed to knock down CD9P-1 expression. HEK/CD9P-1 cells were transfected by CD9P-1 oligonucleotide for RNA interference. The expression level was checked by flow cytometry 48h after treatment showing 80% knock down at cell surface. It should be noted that CD9P-1 silencing was stable for more than 72h. Thus, cells were plated on collagen I for 48h after treatment and videomicroscopy experiments were performed. CD9P-1 silencing dramatically decreases cell motility on collagen I of HEK/CD9P-1 as well as HEK-293 (Fig. 3B). Migration of HEK-293 and HEK/CD9P-1 cells was also investigated using Boyden chamber assay. An increase of invading HEK/CD9P-1 cells was observed compared to HEK-293 cells when inserts were coated with collagen I (Fig. 3C).

Altogether, these observations demonstrate that CD9P-1 expression can dramatically change cell migration.

### CD9P1-induced cell motility on collagen I involves α2β1 integrin

The mechanisms underlying CD9P-1 expression's effect on cell migration were investigated. The involvement of β1-integrins which play a role in cell adhesion and migration as receptors of extracellular matrices [42] was addressed. We focused on the mechanism underlying cell migration on collagen I because CD9P-1 expression induced an increase of cell motility on this matrix. The use of the blocking antibody 4B4 showed that cell motility of HEK-293 and HEK/CD9P-1 cells is dependent on β1-integrins because cells were no longer migrating on collagen I when the antibody was added to the culture media (data not shown).

**Figure 4:**
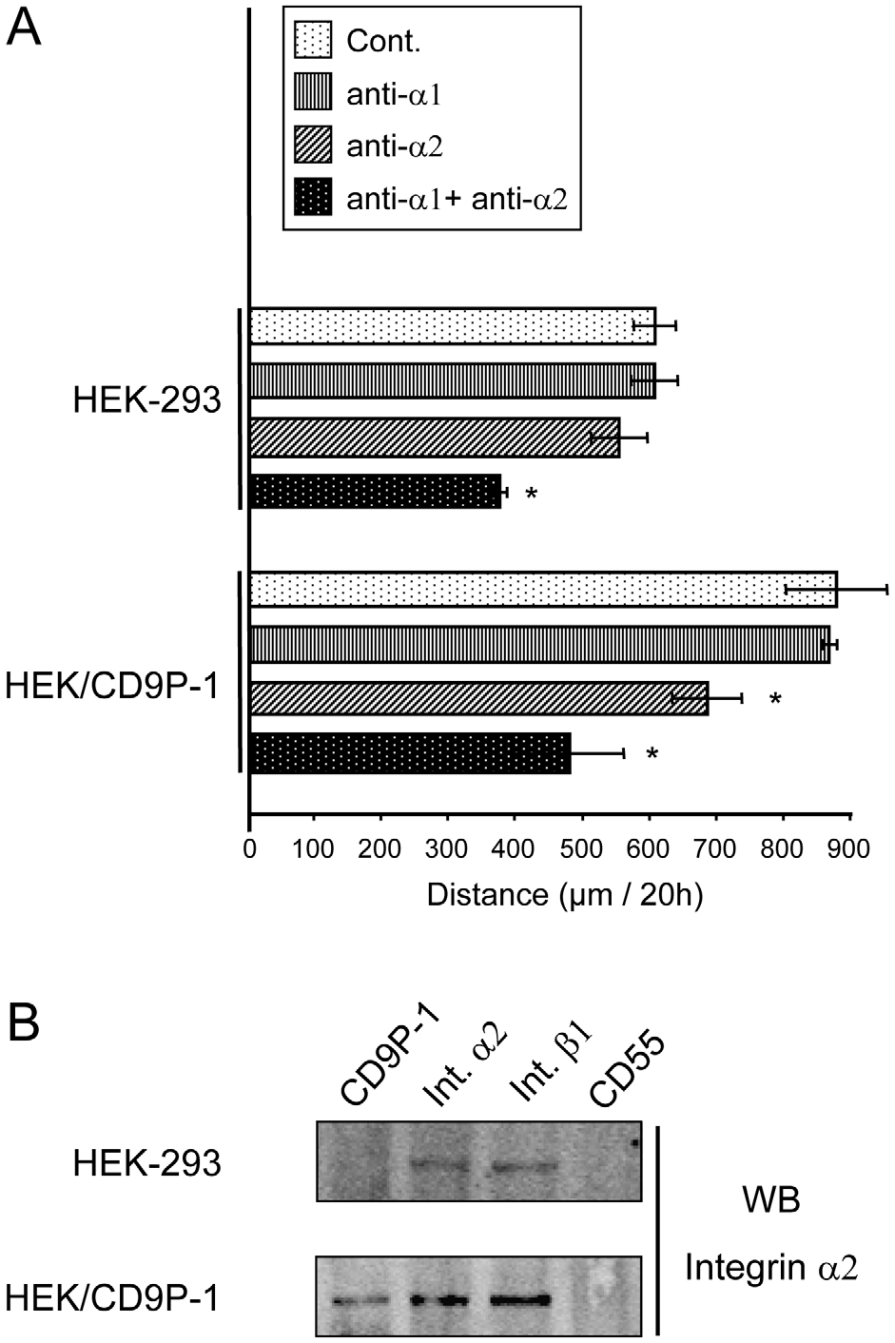
Functional and physical link between CD9P-1 and α2β1 integrin. (A) Time-lapse videomicroscopy experiments were performed with HEK-293 or HEK/CD9P-1 cells. Monoclonal antibodies directed against integrins α1 (FB12) or α2 (Gi9) were added in the media (10 µg/ml). Independent experiments were performed (n = 6); standard errors are shown; (*) p<0.01. (B) Cells were lysed using the mild detergent Brij58 and immunoprecipitation experiments were performed with mAbs directed against CD9P-1 (1F11), α2-integrin (Gi9), β1-integrin (9D6) and CD55. After electrophoresis and transfer to a membrane, Western blotting was performed using α2-integrin antibody H-293.

The major cellular receptors of the matrix collagen I are the integrins α1β1 and α2β1 [42]. Thus, possible involvement of these integrins was investigated using blocking antibodies. Cell motility of HEK-293 cells on collagen I was not influenced when adding α1-integrin or α2-integrin blocking antibodies separately. However, HEK-293 cell motility was significantly inhibited when both antibodies were added to the culture media. These results suggest that cell migration of HEK-293 cells on collagen I can be mostly driven by either integrins α1β1 or α2β1 because when one is inhibited the other may compensate. In contrast, the treatment of HEK/CD9P-1 cells with the α2-integrin blocking antibody decreased cell motility to a similar level as that observed with HEK-293 cells whereas α1-integrin blocking antibody had no effect. When both antibodies α1- and α2-integrins were added in HEK/CD9P-1 cell culture media, cell motility was inhibited as observed with HEK-293 cells (Fig. 4A). These observations suggest that CD9P-1 expression increases cell motility on collagen I through the possible involvement of α2β1 integrin.

A link between CD9P-1 and α2β1 integrin was investigated. HEK-293 and HEK/CD9P-1 cells were lysed using the mild detergent Brij58. Immunoprecipitation experiments were performed with mAbs CD9P-1, α2-integrin, β1-integrin and CD55 as a control. Under these conditions, α2-integrin was shown to be associated with CD9P-1 only in HEK/CD9P-1 cells (Fig. 4B).

### CD9P-1 transmembrane and cytoplasmic domains are required for inducing effects on cell migration

**Figure 5:**
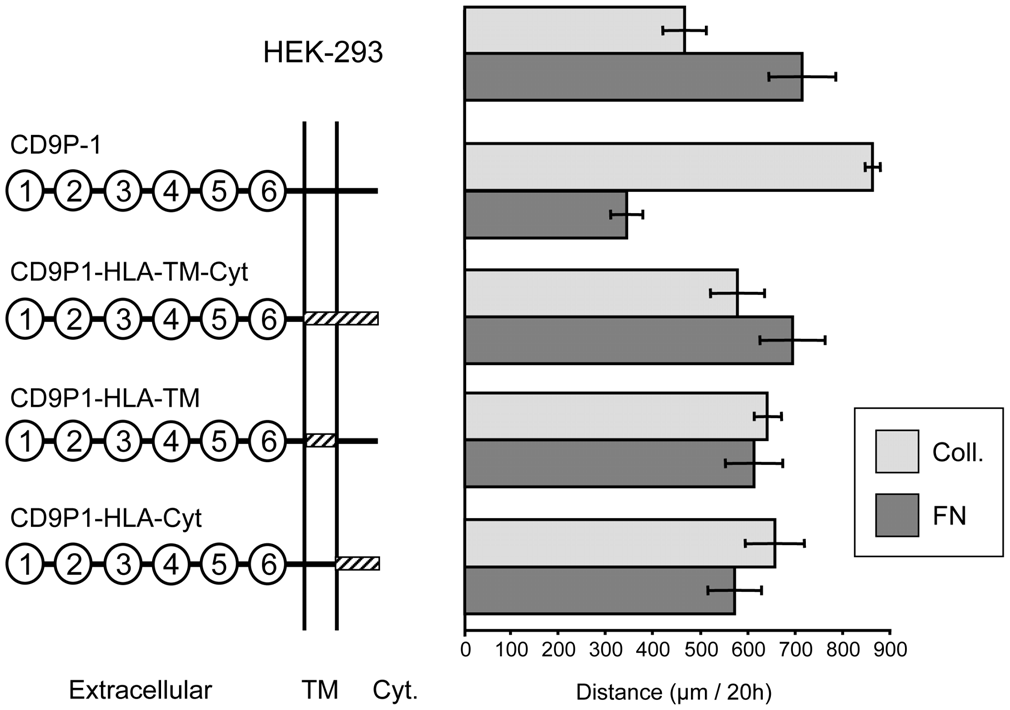
Structure-function relationship of CD9P-1 on cell motility. Wild type CD9P-1 exhibiting six Ig domains, transmembrane domain (TM) and a cytoplasmic domain (Cyt) is represented. CD9P-1 chimeric forms were generated by replacing the transmembrane and/or the cytoplasmic domain by the corresponding domains of HLA class I protein (shaded boxes). Time-lapse videomicroscopy experiments were performed on collagen I or fibronectin with cell lines expressing wild type or chimeric forms of CD9P-1. Three independent experiments were performed; standard errors are shown.

The regions of the CD9P-1 protein involved in cell migration were investigated. Chimeric proteins were generated in which the transmembrane and/or the cytoplasmic domains of CD9P-1 were replaced by corresponding domains from HLA class I. Cell lines stably expressing the chimeric proteins were established using HEK-293 cells [38]. Cells were plated on collagen I or fibronectin. The motility of these cell lines was investigated and compared to HEK-293 and HEK/CD9P-1 using time-lapse videomicroscopy. None of the chimeric proteins were able to promote cell migration on collagen I or to induce its inhibition on fibronectin (Fig. 5). These observations suggest that the transmembrane and the cytoplasmic domains of the CD9P-1 are required for inducing effects on cell migration.

### CD9 and CD81 regulate CD9P1-induced cell motility

**Figure 6:**
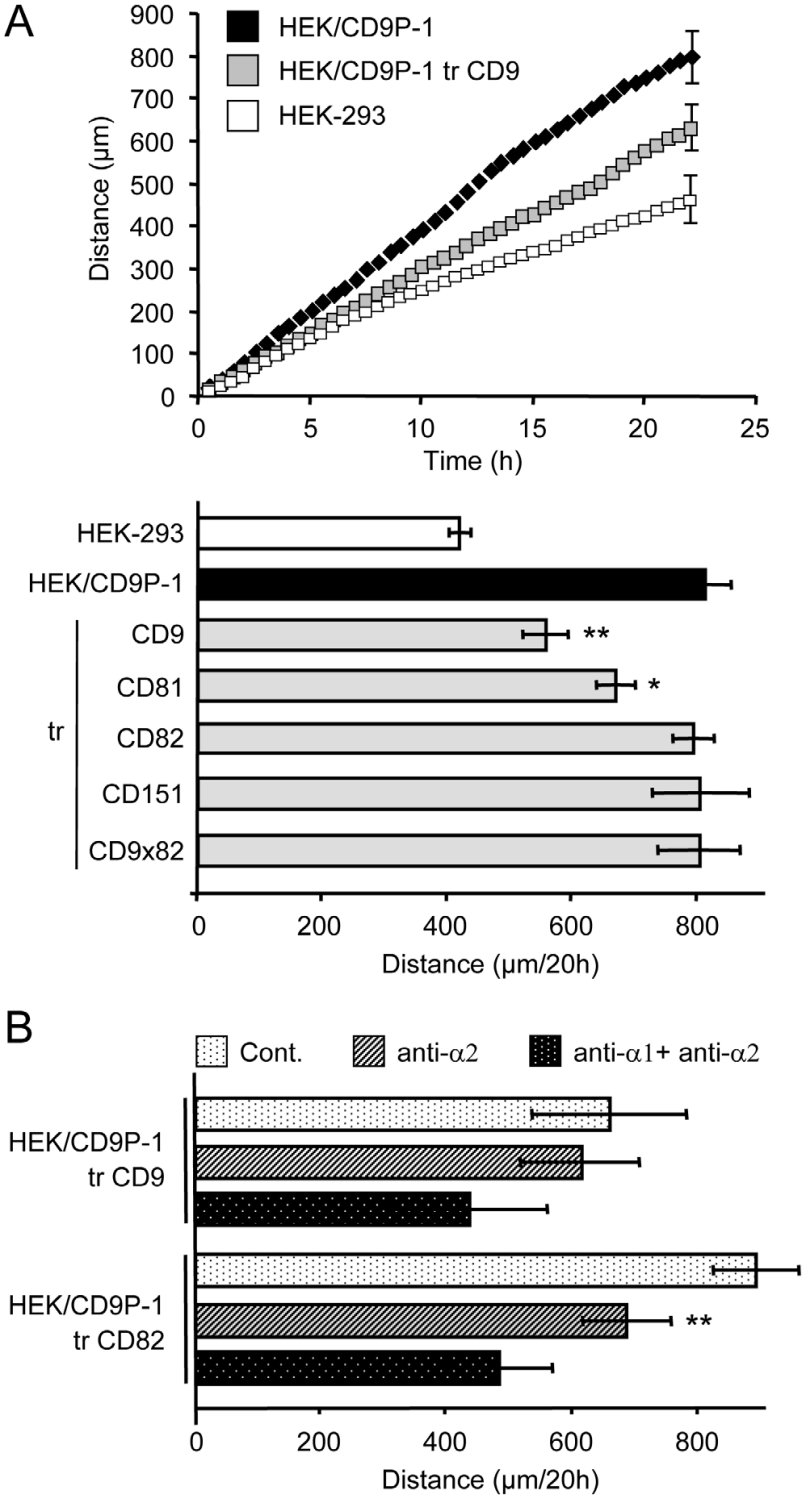
Effects of tetraspanins on CD9P1-induced cell motility on collagen I. (A) HEK/CD9P-1 cells were transfected with tetraspanins CD9, CD81, CD82, CD151 or CD9×82. Then, cells were plated on collagen I. Time-lapse videomicroscopy was performed at a rate of 1 frame per 15 min for 24h. The results correspond to six independent experiments; standard errors are shown. (B) Time-lapse videomicroscopy experiments were performed with HEK/CD9P-1 cells transfected with CD9 or CD82. The mAbs directed against integrins α1 (FB12) or α2 (Gi9) were added to the media (10 µg/ml). Three independent experiments were performed; standard errors are shown. (*) p<0.05, (**) p<0.01.

The effect of tetraspanins on CD9P1-induced cell motility was investigated because CD9P-1 is a major component of the tetraspanin microdomains [15], [21], [24]. HEK/CD9P-1 cells were transfected with plasmids encoding CD9, CD81, CD82 or CD151. After 24h, similar expression levels of the transfected proteins were observed at cell surface, using flow cytometry (Table 1). Cells were plated on collagen I for 24h after transfection and time-lapse videomicroscopy experiments were performed. An increase of CD9 or CD81 expression significantly decreased HEK/CD9P-1 cell motility on collagen I whereas CD82 or CD151 expression had no effect (Fig. 6A). The effect of tetraspanins on the migration properties of HEK/CD9P-1 cells was also investigated using Boyden chamber assay. An increase of CD9 expression decreased the number of invaded cells when inserts were coated with collagen I. As a control, the increased expression of CD82 had no effect (data not shown).

The specific effect observed only with tetraspanins CD9 or CD81 suggested a mechanism mediated through a direct interaction with CD9P-1. Indeed, CD9P-1 specifically associates with tetraspanins CD9 or CD81 to constitute robust primary complexes [15], [21], [24]. To confirm the importance of these primary complexes, the effect on cell motility of the chimera protein CD9×82 which does not associate with CD9P-1 [24] was investigated. Interestingly, the chimera protein CD9×82 had no effect on HEK/CD9P-1 cell motility (Fig. 6A).

The effect of tetraspanin expression on α2β1-dependent cell motility was investigated. Interestingly, HEK/CD9P-1 cells transfected with CD9 were no longer sensitive to the α2-integrin blocking antibody. As a control, CD82 expression had no effect on the sensitivity of HEK/CD9P-1 cells to blocking antibodies (Fig. 6B). It should be noted that CD9 was not observed to associate with integrin α2β1 by immunoprecipitation experiments after cell lysis using the mild detergent Brij58 (data not shown).

**Figure 7:**
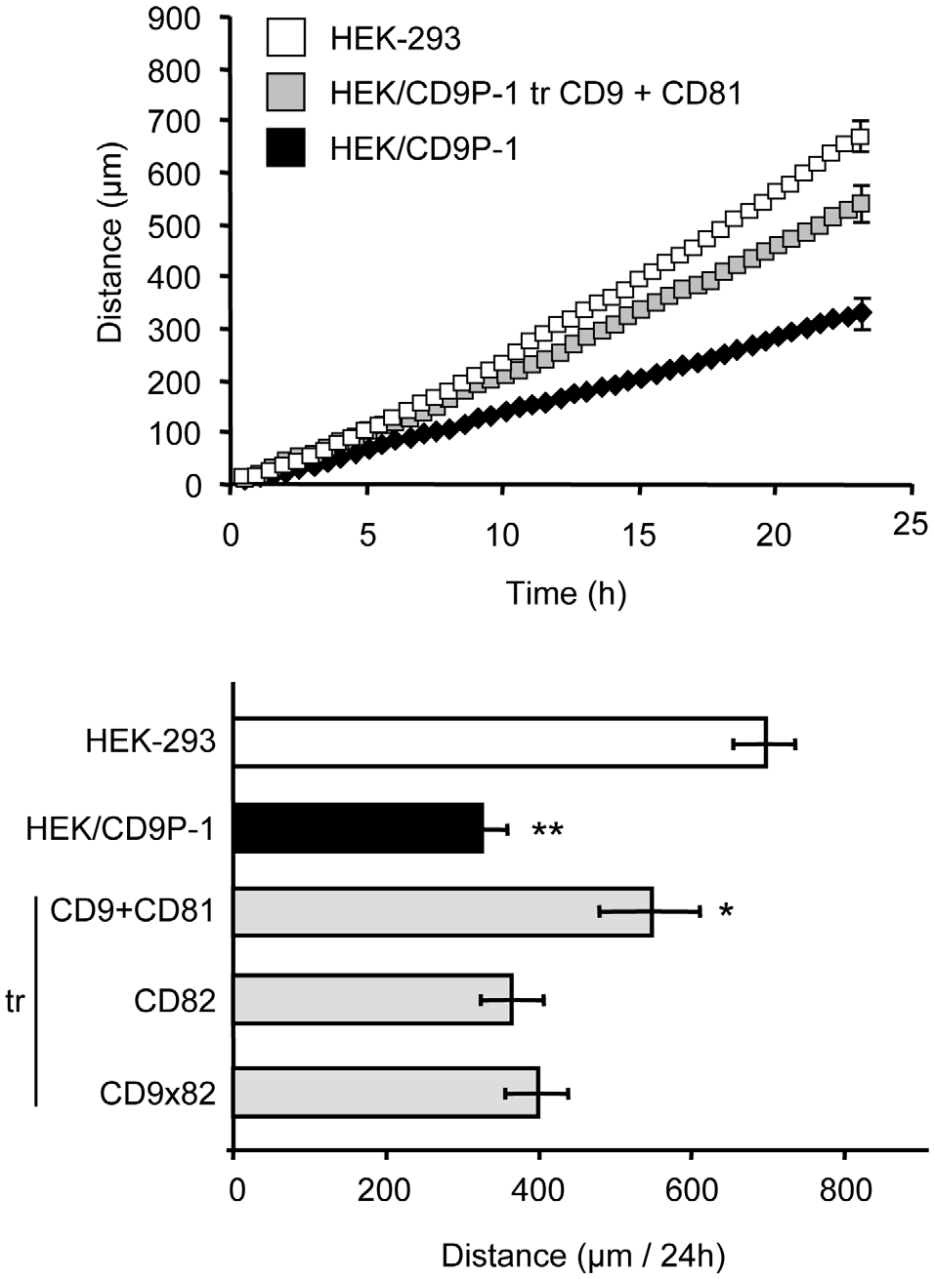
Effects of tetraspanins on CD9P1-induced cell motility on fibronectin. HEK/CD9P-1 cells were transfected with tetraspanins CD9, CD81, CD82 or CD9×82. Then, cells were plated on fibronectin. Time-lapse videomicroscopy was performed at a rate of 1 frame per 15 min for 24h. The results correspond to three independent experiments; standard errors are shown. (*) p<0.05, (**) p<0.01.

Additional experiments were performed on fibronectin. On this matrix, CD9 and CD81 expression were observed to reverse the effects induced by CD9P-1 expression on cell migration (Fig. 7).

In total, these results suggest that CD9 or CD81 can regulate the functional effect of CD9P-1 on cell motility, which may be related to a direct interaction between these tetraspanins and their molecular partner.

### CD9 or CD81 effects on cell motility are related to the level of association with CD9P-1

**Figure 8:**
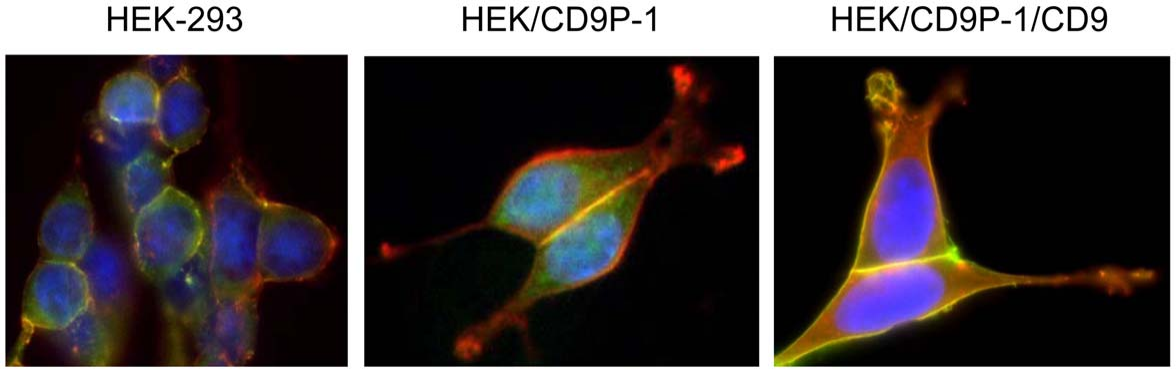
*In situ* labeling of CD9P-1 and CD9. HEK-293, HEK/CD9P-1 and HEK/CD9P-1 transfected (tr) with CD9 were plated on collagen I and cultured in labtek chambers. After 24h, cells were fixed and *in situ* labeling was performed using CD9P-1 mAb (1F11, IgG1) and CD9 mAb TS9b (CD9, IgG2b). Secondary antibodies, anti-IgG1 coupled to Alexa 568 and anti-IgG2 coupled to Alexa 488, were used for differential staining. Chromatin was also stained using 300 nM DAPI. Images were acquired using optical microscopy with epifluorescence at the magnification 630×. Red: CD9P-1; green: CD9; blue: nucleus.

**Figure 9:**
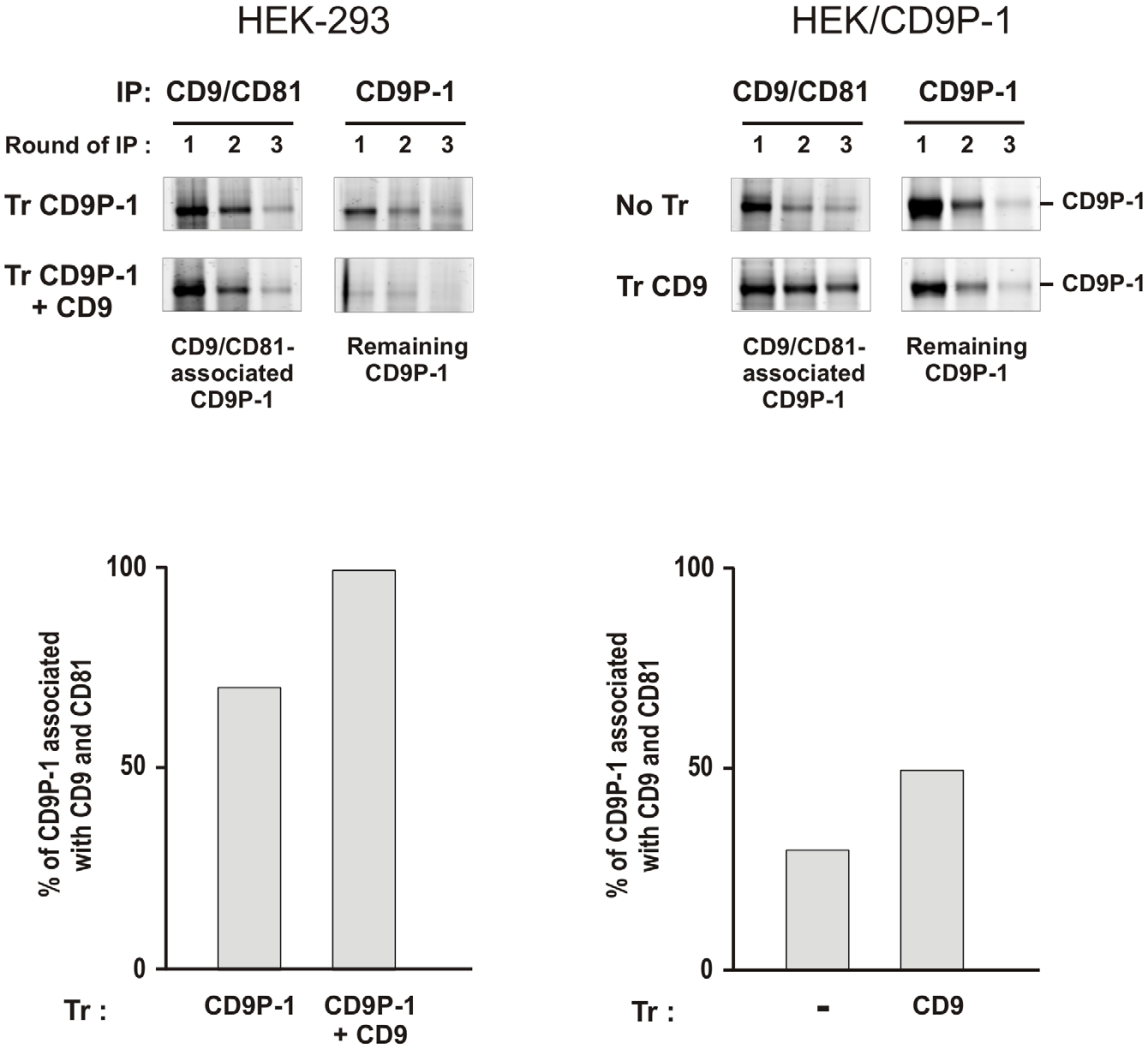
Quantification of the amount of CD9P-1 associated with tetraspanins. The amount of CD9P-1 associated with tetraspanins or that remaining outside the tetraspanin complexes was quantified in HEK-293 or HEK/CD9P-1 cells after transfection with CD9P-1 or/and CD9. Cells were lysed using the mild detergent Brij97. CD9P-1 associated with tetraspanins was investigated by depletion of tetraspanins from the lysate. Three rounds of subsequent immunoprecipitation were performed with mAbs directed against CD9 and CD81. After depletion of tetraspanin complexes, the amount of CD9P-1 still remaining in the lysate and therefore not associated with tetraspanins was measured by three additional rounds of immunoprecipitation using a mAb directed against CD9P-1 (1F11) antibody. All immunoprecipitates were separated by electrophoresis under reducing conditions and proteins were stained using colloidal Coomassie blue. Gels were scanned using the Odyssey device (upper panel). Signals obtained from CD9P-1 were measured and quantitated. When HEK-293 cells were transiently transfected with CD9P-1 (mfi = 300 *versus* 50 from endogenous), more than 70% of CD9P-1 was associated with tetraspanin partners CD9 (mfi = 150) and CD81 (mfi = 300). Furthermore, HEK-293 cells were transfected with both plasmids encoding CD9P-1 and CD9. Under these conditions when CD9 expression level was higher (mfi = 700), we observed that 100% of CD9P-1 was associated with tetraspanins. In HEK/CD9P-1 cells that stably expressed CD9P-1 with a high level (mfi = 1000), the amount of CD9P-1 associated with tetraspanins was estimated to be 30%. When HEK/CD9P-1 cells were transfected with CD9 (mfi = 700), a higher amount, corresponding to 50% of CD9P-1, was associated with tetraspanins CD9 and CD81 (lower panel). A representative experiment is shown. IP: immunoprecipitation; Tr: transfection.

The correlation between the level of association of CD9P-1 with its tetraspanin partners and cell motility was investigated. The sub-cellular distributions of CD9P-1 and CD9 were investigated by *in situ* labelling of HEK-293 and HEK/CD9P-1 cells. As expected, CD9 and CD9P-1 exhibited expression at the plasma membrane and were co-localized in HEK-293 cells. CD9P-1 was also observed to be highly expressed at the plasma membrane on HEK/CD9P-1 cells. However, CD9 and CD9P-1 were co-localized only at cell to cell contact in these cells on collagen I. Indeed, a high signal corresponding to CD9P-1 was observed in structures such as pseudopodia that did not contain any detectable CD9 (Fig. 8). Although CD9P-1 associates specifically with CD9, these observations suggest that some CD9P-1 can remain out of tetraspanin microdomains in HEK/CD9P-1 cells. Furthermore, CD9 was overexpressed in HEK/CD9P-1 cells after transfection (HEK/CD9P-1/CD9). Under these conditions, the increased expression of CD9 led to the restoration of a complete co-localization of CD9P-1 and CD9 (Fig. 8). The amount of CD9P-1 associated with tetraspanins was further quantified by immunoprecipitation experiments using tetraspanin mAbs following cell lysis with the mild detergent Brij97 which has been demonstrated to preserve tetraspanin complexes [15]. A good correlation was observed between CD9 expression and the level of CD9P-1 association with tetraspanins (Fig. 9). These results suggest that the ratio of expression levels between CD9P-1 and its tetraspanin partners regulate cell motility.

## Discussion

This is the first report addressing the function of the membrane protein CD9P-1. We have demonstrated that CD9P-1 expression induces dramatic changes in cell spreading and motility in HEK-293 cells. Interestingly, the functional effects related to CD9P-1 expression appeared to be dependent of matrices such as collagen I or fibronectin. Indeed, CD9P-1 plays a role as activator of cell motility on collagen I, whereas it was observed to be an inhibitor on fibronectin. CD9P-1 belongs to a new family of membrane proteins with Ig domains that includes CD101, IgSF3 and EWI-2 [9]. Overexpression of EWI-2 in A431 epidermoid carcinoma cells has been demonstrated to impair cell reaggregation and motility functions on laminin-5 [43]. In the T leukaemia cell line Molt-4, overexpression of EWI-2 markedly impaired spreading and ruffling on VCAM-1 [44]. Furthermore, a mutant EWI-2 molecule with the cytoplasmic tail replaced by that of CD2 neither impaired neither cell spreading nor migration [43], [44]. The generation of CD9P-1 and HLA class I chimeric proteins demonstrated that both the transmembrane and cytoplasmic domains of CD9P-1 are involved in cell motility. Although the CD9P-1 cytoplasmic domain is small, at only 26 aa, it appears to be crucial in the activation of cell motility. This domain may activate cell motility though its intracytoplasmic domain by associating with the cytoskeleton. Indeed, CD9P-1 has been demonstrated to associate with ERM (Ezrin, Radixin and Moesin) [45]. On the other hand, the cytoplasmic domain contains a consensus phosphorylation site (RXRXXS/T) [46] on serine 875 (RRRLMS). In a recent proteomic study, serine 875 has been identified by mass spectrometry as being phosphorylated [47]. Thus, CD9P-1 may be involved in signaling upstream adhesion molecules such as integrins.

CD9P-1 induced effects on cell motility are related to β1-integrins as demonstrated by the use of blocking mAbs which completely stopped cell migration. On collagen I, the increase of cell motility may be related to integrin α2β1. With this regard, CD9P-1 has been shown to associate with integrin α2β1. However, the association between CD9P-1 and integrin α2β1 has only been observed using the mild detergent Brij58. This suggests that activation of cell motility through integrin α2β1 is most likely indirect and may necessitate other membrane proteins. In addition, cross-linking experiments performed on living cells did not show a direct interactions between those two membrane proteins (data not shown). Therefore, it would be of great interest to identify CD9P-1 associated proteins that may be involved in the crosstalk between CD9P-1 and integrins as well as in signaling upstream such molecules.

This study has suggested that tetraspanin microdomains can play a role in the modulation of cell motility by the regulation of CD9P-1 compartmentalization. Indeed, CD9P-1 exhibits a dramatic effect on cell motility when located outside of tetraspanin microdomains. The amount of CD9P-1 associated with tetraspanins has been precisely quantified by subsequent immunoprecipitations and measuring by infrared fluorescence on stained gels [38]. In HEK-293 cells, tetraspanin microdomains contain the total amount of CD9P-1. When CD9P-1 is overexpressed, a fraction of CD9P-1 can localize to other membrane compartment, as demonstrated by cell labeling and biochemical experiments. These observations suggest a saturation of the tetraspanin microdomains. Moreover, variation in the expression levels of tetraspanins CD9 or CD81 induces a change in the fraction of CD9P-1 contained in tetraspanin microdomains. It is important to note that relocation of CD9P-1 into tetraspanin microdomains is mediated by direct interaction with CD9 or CD81 because other tetraspanins such as CD82 and CD151 which do not associate directly with CD9P-1 had no effect (data not shown). Furthermore, CD9 or CD81 expression has been shown to reverse CD9P-1 induced effects on cell motility with a concomitant association with CD9P-1. These observations strengthen the impact of the expression level of tetraspanins on membrane compartmentalization of their partners and the functional relevance of such microdomains. In pathological conditions such as cancer, variation in the expression level of CD9 or CD81 may induce dramatic effects on cell motility through a change in the membrane compartmentalization of CD9P-1, thus favoring metastasis.
